# Completeness of Communicable Disease Reporting, North Carolina, USA, 1995–1997 and 2000–2006

**DOI:** 10.3201/eid1701.100660

**Published:** 2011-01

**Authors:** Emily E. Sickbert-Bennett, David J. Weber, Charles Poole, Pia D.M. MacDonald, Jean-Marie Maillard

**Affiliations:** Author affiliations: University of North Carolina Health Care System, Chapel Hill, North Carolina, USA (E.E. Sickbert-Bennett, D.J. Weber);; University of North Carolina, Chapel Hill (E.E. Sickbert-Bennett, D.J. Weber, C. Poole, P.D.M. MacDonald);; North Carolina Department of Health and Human Services, Raleigh, North Carolina, USA (J.-M. Maillard)

**Keywords:** Infectious disease surveillance, surveillance, notifiable diseases, communicable diseases, bacteria, viruses, disease notification, epidemiology, research

## Abstract

Despite widespread use of communicable disease surveillance data to inform public health intervention and control measures, the reporting completeness of the notifiable disease surveillance system remains incompletely assessed. Therefore, we conducted a comprehensive study of reporting completeness with an analysis of 53 diseases reported by 8 health care systems across North Carolina, USA, during 1995–1997 and 2000–2006. All patients who were assigned an International Classification of Diseases, 9th Revision, Clinical Modification, diagnosis code for a state-required reportable communicable disease were matched to surveillance records. We used logistic regression techniques to estimate reporting completeness by disease, year, and health care system. The completeness of reporting varied among the health care systems from 2% to 30% and improved over time. Disease-specific reporting completeness proportions ranged from 0% to 82%, but were generally low even for diseases with great public health importance and opportunity for interventions.

## Abstract 2

Reporting proportions were <50% for 49 of the 53 diseases evaluated.

Surveillance has been the cornerstone of public health since the US Congress authorized the Public Health Service to collect morbidity data for cholera, smallpox, plague, and yellow fever in 1878. Currently, all states conduct notifiable disease surveillance following guidelines from the Centers for Disease Control and Prevention (CDC) and the Council for State and Territorial Epidemiologists. The current list of nationally notifiable communicable diseases has expanded to >60 diseases and includes vaccine-preventable diseases (e.g., pertussis, measles), emerging infectious diseases (e.g., severe acute respiratory syndrome, West Nile virus encephalitis), foodborne diseases (e.g., Shiga toxin–producing *Escherichia coli* and *Salmonella* spp. infections), sexually transmitted diseases (e.g., syphilis, HIV), and aerosol and droplet transmitted diseases (e.g., tuberculosis, meningococcal meningitis). Active surveillance programs conducted by CDC in conjunction with certain states include Active Bacterial Core surveillance, FoodNet, and influenza-related hospitalization surveillance. Surveillance of epidemiologically important diseases provides critical information to clinicians and public health officials for use in measuring disease incidence in communities, recognizing disease outbreaks, assessing prevention and control measure effectiveness, allocating public health resources, and further clarifying the epidemiology of new and emerging pathogens (*1*).

Like all US states, North Carolina has state laws and regulations mandating communicable disease reporting (*2**–**4*). The state relies on physicians and laboratories to comply with the directive to report diseases and laboratory results indicative of diseases considered a threat to public health. During the periods of this study (1995–1997, 2000–2006), mandatory reporting was required for >60 diseases. Conditions and disease reports consisted of paper communicable-disease report forms that contained demographic, clinical, and risk factor data for the case-patient. These reports were required to be submitted to the health department within a specified period (i.e., immediately, within 24 hours, or within 7 days), depending on the disease. An important change to the communicable disease surveillance system of the North Carolina Department of Health and Human Services (NC DHHS) occurred when the state administrative code was amended in September 1998 to require that persons in charge of diagnostic laboratories report positive laboratory results for most diseases already reportable by physicians (*2*). This dual reporting mechanism was intended to improve completeness, timeliness, and accuracy of surveillance. More recently, in 2002, surveillance efforts have also expanded with the introduction of 7 regional public health teams and 11 hospital-based public health epidemiologists.

Despite the widespread use of these surveillance data, systematic data collection based on mandatory physician and laboratory reporting has never been extensively evaluated. To date, only 2 evaluations have examined reporting proportions for >5 diseases (*5**,**6*). Previous studies examining the completeness of disease reporting have differed considerably in terms of the following factors: size of geographic region (e.g., from clinics at a single university to multiple states), range of study period (e.g., several months to several years), heterogeneity of reporting systems (e.g., health care provider–based passive reporting vs. health care provider– and laboratory-based passive reporting), and various patient ascertainment methods (e.g., laboratory records, billing records, active surveillance, death certificates). This variability renders study results difficult to compare or aggregate. Therefore, we undertook a comprehensive study of reporting completeness with an analysis of 53 reportable diseases and conditions in selected health care systems across North Carolina over a 10-year period to estimate disease-specific reporting proportions, describe changes to reporting over time, and examine the variability of reporting completeness between health care facilities.

## Methods

A retrospective cohort study was conducted at 8 large nonfederal acute care health care systems that experience 32% of all inpatient visits and 23% of all outpatient visits in North Carolina (*7*). These health care systems ranged in size from 581 to 1,324 site-licensed beds, spanned the Eastern Coastal, Central Piedmont, and Western Mountain regions of the state, and were selected from a network of 11 health care systems staffed with hospital-based public health epidemiologists. The study cohort was defined as all inpatients and outpatients at the 8 health care systems who were assigned a discharge diagnostic code from the International Classification of Diseases, 9th Revision, Clinical Modification (ICD-9-CM), that corresponded with a reportable communicable diseases during a 10-year time period (1995–1997, 2000–2006). The years 1998–1999 were excluded from the study because this period marked the transition when the state law was changed to include a reporting requirement for laboratories.

Diseases were excluded if they were chronic infectious diseases that resulted in a recurring assignment of ICD-9-CM code (e.g., HIV, hepatitis B carrier), if no specific ICD-9-CM code was available (e.g., for viral hemorrhagic fever), or if the NC DHHS did not record patient identifiers in their surveillance database during the entire study period (e.g., for syphilis, gonorrhea, chlamydia). Approval for the study was granted by the institutional review boards of all health care systems as well as by the North Carolina Division of Public Health because identifiable patient data were required to match the hospital and health department databases.

The cohort of patients assigned ICD-9-CM diagnostic codes by the health care systems for a reportable communicable disease were matched to the NC DHHS reported case-patients by using a unique identifier created by either Social Security number, or a combination of the first 2 letters of the last name, first letter of the first name, date of birth, and a 2-digit disease code. Repeat patient visits within a 31-day window for the same disease were enumerated and only the first visit was retained, with the exception of tuberculosis, which had a 365-day window, and hepatitis A and paralytic polio, which were restricted to only the first visit. Patients who had dates of reporting to the NC DHHS before the date of diagnosis at the health care system were excluded because they represented cases that had already been reported.

Unadjusted disease-specific reporting completeness proportions were calculated by dividing the number of case-patients that were reported to NC DHHS by the total number of patients identified in the health care systems who were assigned an ICD-9-CM diagnostic code for a reportable disease. In addition, completeness proportions were estimated by year (1995–1997, 2000–2006) for the 3 health care systems that had complete data available for all 10 years, and generalized linear regression models were used to examine the time trends. For the years 2000–2006, reporting completeness proportions and 95% confidence intervals (CIs) were estimated for each health care system by using a binomial logistic regression model that included as covariates whether or not specific health care system personnel were designated for disease reporting.

For disease-specific completeness proportions, empirical continuity corrections were used when no patients were reported for a disease (*8*). In addition, adjusted completeness proportions and 95% uncertainty intervals (UIs) were calculated by using semi-Bayesian analysis (*9*) as recommended to reduce the mean squared error when an ensemble of measures are estimated (*10*). This semi-Bayesian hierarchical regression analysis uses prior covariates that help explain the mean of the ensemble of estimates and a specified prior variance (τ^2^) of the distribution. Traditional maximum-likelihood estimates (i.e., unadjusted estimates as presented here) can be viewed as a special case of semi-Bayesian analysis in which the prior variance is infinite. By specifying even a moderately informative prior variance such as a τ^2^ indicating that 95% of all completeness proportions lie between 7.3% and 85%, an appreciable reduction in the overall mean squared error can be expected with a shift in the point estimate and a narrowing of the 95% UI for each completeness proportion, with the relative degree of narrowing being greater for diseases with less information.

A sensitivity analysis was conducted on the specified prior variance, τ2, by using high, medium, and low τ2 values that assumed 95% of the completeness proportions were within the following ranges: 2.2%–95%, 7.3%–85%, and 12.9%–75%, respectively. Sensitivity analyses were also conducted on the inclusion or exclusion of prior covariates, which were the time frame for reporting the disease (i.e., 24 hours vs. 7 days), whether or not the disease had a reportable laboratory result, whether or not the disease had reportable serologic test results, whether or not the disease is classified as a CDC category A bioterrorism agent, and the mode of transmission of the disease (person-to-person, arthropod-borne, food/water-borne, droplet/aerosol).

## Results

Unadjusted and adjusted disease-specific completeness proportions for 2000–2006 with 95% CIs and UIs, respectively, are summarized in the Table. The adjusted disease-specific, completeness proportions ranged from 0% to 82.0%, and almost all diseases (49/53) had completeness proportions <50%. Eleven diseases accounted for 90% of disease reporting: salmonellosis, tuberculosis, meningococcal disease, Rocky Mountain spotted fever, campylobacteriosis, shigellosis, acute hepatitis A, pneumococcal meningitis, legionellosis, malaria, and *Haemophilus influenzae* invasive disease. Some unexpected diseases had cases identified with an ICD-9-CM code; for example, anthrax had 14 cases identified, paralytic polio had 32 cases identified, human rabies had 12 cases identified, and smallpox had 9 cases identified. The most dramatic adjustments in the unadjusted to adjusted point estimates were noted for staphylococcal foodborne disease, and for foodborne diseases caused by *Vibrio vulnificus* and other *Vibrio* spp., with an ≈80% change in point estimate for the latter. However, wide UI reflect the imprecision in these estimates.

**Table:** . Disease-specific reporting completeness proportions in North Carolina, USA, 2000–2006*

Communicable disease	No. reported to NC DHHS	No. identified by ICD-9-CM codes	Unadjusted RCP, % (95% CI)	Semi-Bayesian adjusted RCP, % (95% UI)
Anthrax	0	14	0.01 (0.00–100.00)	0.00 (0.00–100.00)
Arboviral encephalitis	0	18	0.00 (0.00–100.00)	8.67 (0.80–52.77)
Botulism	0	4	0.02 (0.00–100.00)	0.08 (0.00–100.00)
Brucellosis	0	33	0.00 (0.00–100.00)	23.02 (1.36–86.62)
Campylobacteriosis	39	97	40.21 (30.94–50.22)	39.96 (30.82–49.85)
Cholera	0	6	0.01 (0.00–100.00)	18.58 (2.24–69.41)
CJD/vCJD	0	32	0.00 (0.00–100.00)	0.87 (0.03–22.97)
Cryptosporidiosis	10	84	11.90 (6.53–20.73)	12.59 (7.07–21.42)
Cyclosporiasis	0	3	0.03 (0.00–100.00)	18.59 (2.25–69.42)
Dengue	4	25	16.00 (6.14–35.69)	14.48 (5.92–31.31)
Diphtheria	0	5	0.02 (0.00–100.00)	8.28 (0.82–49.70)
*Escherichia coli* infection	1	3	33.33 (4.34–84.65)	24.67 (5.82–63.45)
Foodborne staphylococcal infection	0	14	0.01 (0.00–100.00)	74.74 (16.74–97.76)
Granulocytic ehrlichiosis	0	67	0.00 (0.00–100.00)	8.66 (0.80–52.74)
Hantavirus infection	0	3	0.03 (0.00–100.00)	10.10 (0.62–67.06)
Hemolytic uremic syndrome	5	429	1.17 (0.49–2.77)	2.20 (0.99–4.84)
*Hemophilus Influenzae*	14	1,086	1.29 (0.76–2.16)	1.45 (0.87–2.42)
Hepatitis A	27	866	3.12 (2.15–4.51)	3.34 (2.31–4.81)
Legionellosis	24	98	24.49 (16.99–33.95)	24.04 (16.72–33.27)
Leptospirosis	0	33	0.00 (0.00–100.00)	23.02 (1.36–86.62)
Listeriosis	10	64	15.63 (8.62–26.67)	16.14 (9.12–26.95)
Lyme disease	8	790	1.01 (0.51–2.01)	1.18 (0.60–2.30)
Malaria	17	155	10.97 (6.93–16.94)	10.71 (6.80–16.47)
Measles	0	14	0.01 (0.00–100.00)	15.98 (1.41–71.63)
Meningococcal disease	38	179	21.23 (15.85–27.83)	21.19 (15.85–27.73)
Monocytic ehrlichiosis	1	4	25.00 (3.35–76.22)	14.84 (3.12–48.52)
Mumps	1	96	1.04 (0.15–7.02)	1.07 (0.20–5.49)
Plague	0	28	0.00 (0.00–100.00)	0.00 (0.00–100.00)
Pneumococcal meningitis	20	191	10.47 (6.86–15.67)	10.61 (6.99–15.80)
Polio, paralytic	0	32	0.00 (0.00–100.00)	18.56 (2.24–69.38)
Psittacosis	0	21	0.00 (0.00–100.00)	17.45 (1.57–73.69)
Q fever	3	14	21.43 (7.07–49.43)	25.68 (9.14–54.28)
Rabies, human	0	12	0.01 (0.00–100.00)	59.69 (8.00–96.19)
Rocky Mountain spotted fever	40	986	4.06 (2.99–5.48)	4.19 (3.10–5.66)
Rubella	0	39	0.00 (0.00–100.00)	15.97 (1.41–71.61)
Rubella congenital syndrome	0	10	0.01 (0.00–100.00)	1.08 (0.07–15.32)
Salmonellosis	263	594	44.28 (40.33–48.30)	44.82 (40.87–48.83)
SARS (coronavirus infection)	0	1	0.08 (0.00–100.00)	5.71 (0.28–56.27)
Shigellosis	38	213	17.84 (13.26–23.57)	18.17 (13.56–23.93)
Smallpox	0	9	0.01 (0.00–100.00)	0.00 (0.00–100.00)
Streptococcal infection, group A	8	111	7.21 (3.65–13.75)	7.40 (3.80–13.92)
Tetanus	1	20	5.00 (0.70–28.22)	5.25 (1.09–21.78)
Toxic shock syndrome	4	142	2.82 (1.06–7.26)	3.22 (1.28–7.83)
Trichinosis	0	23	0.00 (0.00–100.00)	20.21 (1.82–77.58)
Tuberculosis	100	1,439	6.95 (5.74–8.38)	7.10 (5.87–8.55)
Tularemia	0	6	0.01 (0.00–100.00)	0.04 (0.00–100.00)
Typhoid, acute	3	12	25.00 (8.28–55.18)	21.57 (7.49–48.30)
Typhus, epidemic (louse-borne)	0	2	0.04 (0.00–100.00)	2.93 (0.12–42.63)
Vaccinia	0	13	0.01 (0.00–100.00)	8.27 (0.82–49.68)
*Vibrio* spp. infection, other	0	1	0.08 (0.00–100.00)	81.58 (20.46–98.71)
*Vibrio vulnificus* infection	0	2	0.04 (0.00–100.00)	81.57 (20.45–98.71)
Whooping cough (pertussis)	11	54	20.37 (11.65–33.16)	20.31 (11.78–32.72)
Yellow fever	0	3	0.03 (0.00–100.00)	8.69 (0.80–52.81)

*NC DHHS, North Carolina Department of Health and Human Services; ICD-9-CM, International Classification of Diseases, 9th Revision, Clinical Modification; RCP, reporting completeness proportions; CI, confidence interval; UI, uncertainty interval; CJD, Creutzfeldt-Jakob disease; vCJD, variant CJD; SARS, severe acute respiratory syndrome.

**Figure 1:**
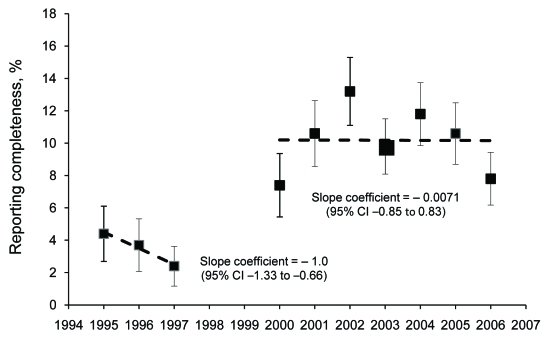
Reporting completeness of communicable diseases in North Carolina, USA, by year, with 95% confidence intervals, 2000–2006.

**Figure 2:**
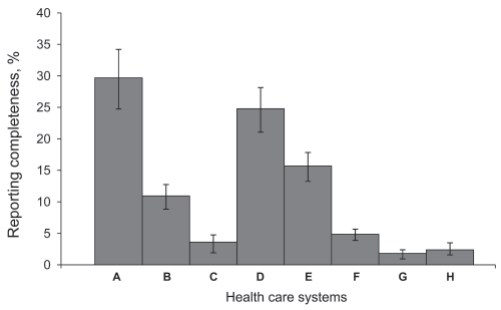
Reporting completeness of communicable diseases in North Carolina, USA, by health care system, 2000–2006. Error bars indicate 95% confidence intervals.

Figure 1 displays the overall reporting proportions by year for the 2 periods, 1995–1997, when only physicians were required to report most diseases, and 2000–2006, when laboratories and physicians were required to report. Reporting increased significantly in the second period, but was still low overall; the linear trend line slope was ≈0 and the intercept was 10.2%. Figure 2 displays the reporting proportions by health care system for the years 2000–2006. The completeness proportions ranged from 1.8% to 29.7% with an overall median proportion of 8.0%. The covariates that described whether or not each health care system designated persons to report had no effect on a health care system’s reporting proportion.

The sensitivity analysis of the τ^2^ values showed that the point estimates and UIs were relatively insensitive to dramatic changes in τ^2^; for example, for meningococcal meningitis with a low τ^2^, the reporting proportion was estimated as 21% (95% UI 16%–28%), with a medium τ^2^, 22% (95% UI 16%–28%); and with a high τ^2^, 22% (95% UI 16%–29%), and the sensitivity analyses examining the use of prior covariates were shown only to have effects on the reporting proportion and 95% UI for diseases with sparse data; for example, cholera with all prior covariates 22% (95% UI 3%–74%), no prior covariates 10% (95% UI 1%–51%), time covariate alone 50% (95% UI 10%–89%).

## Discussion

The public health surveillance system in North Carolina is similar to surveillance systems used nationwide, and, although federal funding in addition to state and local budgets support the infrastructure and maintenance of these systems, they are rarely evaluated with respect to the completeness of the communicable disease data reported. North Carolina’s size (ranked 11th in the 2000 US Census) and population diversity enabled a thorough evaluation of the completeness of reporting many reportable communicable diseases that have rarely been evaluated in previous studies.

Disease-specific reporting completeness proportions were estimated to be low and varied greatly according to disease. Notably, even for diseases that require immediate public health intervention, we found that a low proportion of cases were reported to the health department (e.g., meningococcal meningitis 21.2%, pertussis 20.3%). Further research studies should be undertaken to focus on methods to improve completeness and timeliness of case reporting, especially for these diseases that are severe and require immediate public health intervention.

Variations in disease reporting can occur for several reasons. First, clinicians may have the perception that some diseases are a greater public health threat based on communicability or severity of the illness and the likelihood of death (e.g., tuberculosis vs. salmonellosis). Second, some diseases have relatively straightforward and primarily laboratory-based case definitions (e.g., stool culture positive for *Salmonella* spp. infections with a clinically compatible illness), whereas others are more complex, either requiring multiple laboratory results (e.g., 4-fold increase between acute-phase and convalescent-phase serologic results for Rocky Mountain spotted fever) or a combination of multiple clinical signs and symptoms without any specific laboratory result (e.g., toxic shock syndrome which, requires the presence of at least 4 symptoms). One clear pattern that emerged in our findings was that diseases with fewer clinical criteria and laboratory-based case definitions tended to have higher reporting rates (e.g., salmonellosis 44.8% vs. toxic shock syndrome 3.2%). Laboratory-based case definitions ensure that a dual reporting system exists, and the process is more straightforward because less time is required for reviewing medical records for clinical signs and symptoms. This finding underscores the need for simplicity of case definitions, an essential attribute in surveillance system development and maintenance. Future research on predictors for reporting completeness would be useful for designing interventions to improve reporting and for guiding the future direction of surveillance.

Notably, we identified some patients by ICD-9-CM diagnostic codes for some diseases known to be eliminated in the United States (e.g., smallpox and polio) and others that were highly unlikely to have occurred (e.g., anthrax and human rabies). Numerous previous studies that have evaluated reporting completeness have also used ICD-9-CM codes (*5**,**6**,**11*) because they are standard codes that can be queried relatively easily and should capture clinical cases of disease regardless of laboratory confirmation. The accuracy of the ICD-9-CM codes was a potential limitation in our study. Therefore, we also conducted a separate validation study of the positive predictive values of ICD-9-CM codes for communicable disease surveillance by using as the standard a complete medical record review and concordance with published CDC case classification criteria (*12*). These results showed that for most diseases with higher incidence and relatively straightforward diagnoses, the positive predictive values (PPVs) were high (>80%) with the exception of tuberculosis, which had a PPV of 29% (*13*). For diseases with low PPVs, the estimates we present here are likely to be underestimates of the true reporting completeness because the completeness proportion denominator, or the number of patients identified by ICD-9-CM codes for reportable diseases, is likely to be an overestimate (i.e., contain false-positive cases). However, an additional limitation of this study was that we were unable to assess the sensitivity of ICD-9-CM codes (i.e., false-negative cases) for communicable disease reporting. Quantification of the sensitivity and PPVs of ICD-9-CM codes for communicable disease surveillance is essential in the interpretation of all ICD-9-CM data because these codes are used frequently for research studies and have been proposed as adjuncts to electronic, automated surveillance systems.

Bayesian analyses have been shown in theory, simulation, and prediction problems to offer better estimates for measures as varied as baseball batting averages (*14*) and toxoplasmosis prevalence (*15*). We believe that the semi-Bayesian adjusted estimates offer improved overall accuracy for our ensemble of reporting completeness estimates. For example, for completeness proportions where the maximum-likelihood estimation methods result in 0% proportions, it is unlikely that the true proportion is actually 0%. The use of semi-Bayesian methods enables us to incorporate additional prior covariate data to produce results that are likely better and more plausible than maximum-likelihood estimation results. However, for estimates that were based on less information, we still observed wide UIs around the adjusted estimates. Specifically, we did note a dramatic shift in the reporting completeness proportions after semi-Bayesian adjustments for several diseases, including staphylococcal foodborne disease and *V. vulnificus* and other *Vibrio* spp. infections. This shift reflects the imprecision in each disease’s measured estimates of reporting completeness and the adjustment or shrinkage of their proportions to the mean of the prior covariate probability groups (i.e., food/water-borne transmission, and reporting time of 24 hours). These estimates are shrunk toward the mean of the food/water-borne transmission group of diseases which includes many of those with the highest reporting proportion (e.g., campylobacteriosis, salmonellosis). This finding reinforces the importance of careful specification of prior covariates as well as judicious examination and interpretation of the unadjusted and semi-Bayesian adjusted estimates along with their precision.

The reporting variation seen among health care systems (Figure 2) may be explained in part by health care systems’ internal policies that assign the responsibility for communicable disease reporting to the infection prevention department. For example, the health care system with the highest reporting proportion (health care system A) has hospital-based public health epidemiologists or infection preventionists responsible for disease reporting, and the health care system with the lowest reporting proportion (health care system G) does not assign any additional reporting responsibility beyond the state-mandated reporting by physicians and laboratories. However, adjusting for these health care system policies did not modify the reporting completeness proportions. Currently, the North Carolina general statute states that medical facilities may report (*16*) as opposed to physicians and persons in charge of laboratories who shall report (*17**,**18*). Because infection preventionists typically receive laboratory data daily, are well-trained on case definition application, and share disease prevention goals with the health department, they can serve as partners to the local health department in ensuring that diseases are reported and investigated appropriately. However, redundancy in disease reporting responsibilities could also cause reporting fatigue and the mistaken assumption that someone else has reported the case-patient (*19**,**20*). In addition, external generalizations of these findings to other health care systems should be approached with caution because the participating sites were part of an existing network that includes the largest health care systems in North Carolina and therefore may have been more likely to treat patients who had more severe illnesses or who did not receive a diagnosis at a local clinic or smaller hospital.

## Conclusions

The general trend of the yearly reporting completeness proportions suggests that disease reporting has improved over time yet remains low. Several notable changes occurred in North Carolina’s surveillance system during this period. First, in 1998, the inclusion of laboratory-mandated reporting served as a secondary reporting mechanism in addition to the already mandated physician-based reporting. Regional public health teams were established in 2002 to assist health departments with outbreak investigations. In 2003, a network of public health epidemiologists (funded through the state’s Public Health Emergency Preparedness cooperative agreement with CDC) were placed in hospitals to facilitate disease reporting and case investigation, and, also in 2003, a statewide emergency department–based syndromic surveillance system (North Carolina Disease Event Tracking and Epidemiologic Collection Tool) was created for early case identification. Despite the likely positive effects of these regulatory and programmatic changes on disease reporting, the proportion of diseases reported remains low, as is consistent with data from other passive reporting surveillance systems (21).

More recently, automated alerting and data collection for case-patients with reportable diseases (e.g., a positive blood culture result with gram-negative diplococci triggers an alert with case-patient contact information to infection preventionists, local health department staff, or both) has been shown to increase reporting rates when applied to traditional passive surveillance systems (*22**,**23*). Although North Carolina, like many states, has developed and implemented an electronic disease surveillance system, the reporting of communicable diseases by local health departments still remains largely passive in that reporting is accomplished by accessing a secure Internet site and entering patient information. Physicians who practice outside local health departments currently use paper-based reporting.

When health information exchange becomes a reality, public health surveillance can benefit significantly by automating processes that currently rely on manual data entry. Disease reporting could be automated by standardized queries directly from the electronic health records for key laboratory results (e.g., positive acid-fast bacillus sputum smear) and for simplified or proxy clinical case definitions by using ICD-9-CM diagnosis codes or free-text admission diagnoses. Upon recognition of these potential case-patients, automating surveillance data collection directly from electronic health records to populate data fields for basic patient demographics and laboratory results could also reduce administrative time for physicians and health department officials and expedite communicable disease investigations.

This type of automated technology for electronic health records is consistent with The American Recovery and Reinvestment Act of 2009, which authorizes the Centers for Medicare and Medicaid Services to provide reimbursement incentives for health care entities who are “meaningful users” of certified electronic health record technology. In fact, the recent draft recommendations for defining “meaningful use” from the Health Information Technology Policy Council to the National Coordinator propose that hospitals be capable of providing electronic submission of reportable laboratory results to public health agencies by 2011 (*24*). Such an undertaking will require implementation of national laboratory reporting standards for hospitals and can only be accomplished with resource allocation and partnerships between health departments and health care systems. Furthermore, additional surveillance research should investigate the sensitivity, specificity, and feasibility of using different key laboratory results and proxy clinical case definitions (e.g., ICD-9-CM codes) for automating the identification of potential case-patients. The “meaningful use” of the electronic health record for automated case-finding and data collection will transition our current public health surveillance system from passive to active and thereby overcome the major barriers to complete, accurate and timely communicable disease reporting and surveillance.
